# Effect of chronic stress on human endocrine function: A biomarker-based study

**DOI:** 10.6026/973206300220056

**Published:** 2026-01-31

**Authors:** Ajeet Singh, Pramod Kumar, Hemali Jha, Amrit Podder, Parth Jani

**Affiliations:** 1 Department of Physiology, Santosh Medical College, Ghaziabad, Uttar Pradesh, India; 2 Department of Internal Medicine, Integral Institute of Medical Sciences and Research, Integral University, Lucknow, Uttar Pradesh, India; 3 Department of Physiology, KMC Medical College & Hospital, Maharajganj, Uttar Pradesh, India; 4 Department of General Medicine, Government Medical College, Bhavnagar, India

**Keywords:** Cortisol, endocrine dysfunction, hormonal changes, stress biomarkers, thyroid-stimulating hormone

## Abstract

The impact of chronic stress on endocrine function, particularly its effects on cortisol, thyroid hormones, and sex hormones is of interest. The study employed a cross-sectional design, involving 120 participants (aged 18-60) exposed to chronic stress for 6 months or longer. Elevated cortisol levels and disruptions in thyroid and reproductive hormones were observed, particularly in those experiencing high stress. Data show the potential of biomarkers like thyroid-stimulating hormone (TSH) for monitoring stress-related endocrine dysfunctions. Thus, we show the significant role of chronic stress in hormone dysregulation and its associated health risks. The study offers insights into early detection and therapeutic strategies for stress-related health issues.

## Background:

Chronic stress is a pervasive condition that can significantly impact an individual's overall health. It is commonly understood that stress activates the body's response systems, particularly the endocrine system [1]. This system, which regulates hormones responsible for numerous vital functions, is sensitive to changes in the body's internal and external environments [2]. Under normal conditions, the body experiences stress in short bursts, activating the hypothalamic-pituitary-adrenal (HPA) axis, which triggers the release of cortisol. This hormone helps manage the body's reaction to stress. Once the stressor is removed, cortisol levels return to normal, and the body reestablishes balance [3]. However, when stress becomes chronic, this system can be disrupted. Persistent exposure to stress can lead to prolonged activation of the HPA axis, which in turn causes the continual release of cortisol [4]. Over time, elevated cortisol levels can have adverse effects on various biological functions, including immune system suppression, increased blood pressure, and impaired metabolic processes. Additionally, chronic stress can lead to the dysregulation of other hormones, such as thyroid hormones and sex hormones, further contributing to physical and psychological health issues [5]. The effects of chronic stress on the endocrine system are complex and multifaceted. These disruptions are not only physiological but can also affect mood, cognition, and behaviour, creating a vicious cycle in which stress and its consequences reinforce each other [6]. Hormones such as adrenaline, norepinephrine, and cortisol interact in a delicate balance, and any long-term changes to their levels can lead to serious health complications. Research has shown that chronic stress is associated with a variety of health conditions, including cardiovascular diseases, diabetes, obesity, anxiety disorders, and depression. These conditions are often compounded by hormonal imbalances resulting from sustained stress exposure [7]. Biomarkers play a critical role in understanding the physiological effects of chronic stress on the endocrine system. They can provide measurable indicators of the changes in hormone levels and offer valuable insight into the body's long-term response to stress [8]. By identifying these biomarkers, researchers and healthcare providers can develop more effective diagnostic tools and treatments for individuals with chronic stress [9]. Therefore, it is of interest to determine the specific biomarkers of chronic stress and how they affect endocrine function, providing a deeper understanding of their long-term impact on human health.

## Methodology:

This study aimed to investigate the impact of chronic stress on human endocrine function using a biomarker-based approach. The methodology was designed to collect and analyze data on hormonal changes in individuals exposed to chronic stress. The research employed a combination of quantitative data collection, laboratory testing, and statistical analysis to identify the relationship between chronic stress and endocrine function.

## Study design and population data:

The study followed a cross-sectional design and included a total of 120 participants (60 males and 60 females), aged between 18 and 60 years. Participants were selected from a variety of professional backgrounds and socio-economic statuses to ensure a diverse sample. All participants had been experiencing chronic stress for at least 6 months prior to the study, which was the key inclusion criterion.

## Inclusion criteria:

[1] Adults aged between 18 and 60 years.

[2] Participants who had experienced chronic stress for at least 6 months.

[3] Chronic stress was defined as prolonged exposure to stressors without a significant reduction in perceived stress over time.

## Exclusion criteria:

Individuals with pre-existing endocrine disorders, severe psychiatric conditions or those who were currently on medication that could influence hormonal levels, to ensure that other health factors did not confound the results of the study. The inclusion and exclusion criteria were carefully applied to ensure that the sample accurately represented individuals experiencing chronic stress, while excluding those with medical conditions that could skew the results.

## Chronic stress assessment:

Chronic stress levels in participants were assessed using a combination of subjective and objective measures:

## Subjective measures:

The Perceived Stress Scale (PSS), a widely used psychological instrument, was employed to assess participants' perceived levels of stress over the past month. Participants completed a questionnaire to evaluate various stressors in their daily lives, including work- related stress, family stress, and socioeconomic challenges.

## Objective measures:

Physiological indicators such as heart rate and blood pressure were measured to provide an objective measure of stress at the time of data collection.

## Hormonal analysis:

To evaluate the hormonal changes associated with chronic stress, biological samples were collected from all participants at three points throughout the day (morning, afternoon, and evening). This approach helped capture variations in hormone levels across different times of the day.

## Saliva samples:

These were collected for cortisol measurement, as cortisol levels in saliva are a non-invasive and reliable marker of the body's stress response.

## Blood samples:

Blood was collected to measure key endocrine biomarkers, including:

[1] Thyroid hormones (T3, T4, and TSH) to assess thyroid function.

[2] Sex hormones (testosterone, estrogen) to investigate potential dysregulation due to chronic stress.

## Laboratory testing:

All blood and saliva samples were analyzed in a certified laboratory for the concentration of various hormones. Two techniques were employed for hormone analysis:

High-Performance Liquid Chromatography (HPLC): Used to measure cortisol levels.

Enzyme-Linked Immunosorbent Assay (ELISA): Applied for measuring thyroid and sex hormones due to its high sensitivity and accuracy.

## Data analysis:

The collected data were analyzed using statistical software to identify significant correlations between chronic stress and endocrine biomarkers.

## The analysis involved:

Descriptive statistics: To summarize the demographic characteristics and stress levels of participants.

## Inferential statistics:

Correlation analysis and multiple regression models were used to explore the relationship between chronic stress and hormonal changes. A p-value of less than 0.05 was considered statistically significant to determine meaningful associations between chronic stress and hormone levels.

## Results:

The results of the study were analyzed to examine the impact of chronic stress on human endocrine function, specifically looking at cortisol, thyroid hormones (T3, T4, and TSH), and sex hormones (testosterone and estrogen). The data collected from 120 participants were subjected to statistical analysis to determine any significant correlations between chronic stress and hormonal changes. Table 1 provides the demographic breakdown of the study participants. The sample was evenly split between male and female participants, with a mean age of 34.5 years. Of the 120 participants, 25% reported experiencing low stress, 50% moderate stress, and 25% high stress. Table 2 shows the cortisol levels measured at different times of the day. A significant difference was observed in cortisol levels between morning and evening measurements (p = 0.002). Cortisol levels were highest in the morning, gradually decreasing throughout the day. Table 3 illustrates the thyroid hormone levels (T3, T4, and TSH) in participants with low, moderate, and high chronic stress. The T3, T4, and TSH levels were significantly different between participants with moderate and high stress (p = 0.04 for T4), indicating that higher stress levels were associated with lower thyroid function. Table 4 shows the levels of testosterone and estrogen in participants with varying stress levels. There was a significant decrease in testosterone and estrogen levels in participants with moderate and high stress compared to those with low stress (p = 0.02 for testosterone and p = 0.03 for estrogen), suggesting that chronic stress negatively impacts sex hormone production. Table 5 presents the correlation between chronic stress levels (as measured by the Perceived Stress Scale, PSS) and various hormones. Significant negative correlations were observed between stress and the levels of thyroid hormones (T3 and T4) and sex hormones (testosterone and estrogen), particularly in participants with high stress levels (p < 0.05). The study revealed significant findings regarding the relationship between chronic stress and endocrine function. Cortisol levels were highest in the morning, with a noticeable decline throughout the day (p = 0.002). Participants with high chronic stress showed the greatest reduction in cortisol during the evening, suggesting prolonged stress exposure altered the normal circadian rhythm of cortisol secretion. Regarding thyroid hormones, participants with moderate and high stress levels had lower T3 and T4 levels compared to those with low stress (p = 0.04 for T4), indicating that chronic stress can impair thyroid function. In particular, the TSH levels were higher in participants with high stress, suggesting a potential feedback loop in thyroid regulation due to sustained stress exposure. Sex hormone levels were also affected by chronic stress. Testosterone and estrogen levels were significantly lower in participants with moderate and high stress (p = 0.02 for testosterone and p = 0.03 for estrogen), reflecting the impact of chronic stress on reproductive hormones. Finally, correlation analysis demonstrated strong negative relationships between chronic stress and hormone levels, particularly for cortisol (r = 0.78, p = 0.01), T3 (r = -0.54, p = 0.03), T4 (r = -0.62, p = 0.01), testosterone (r = -0.49, p = 0.04), and estrogen (r = -0.51, p = 0.03). These findings suggest that chronic stress is associated with significant alterations in the endocrine system, which may contribute to a variety of health problems.

## Discussion:

This study's findings provide valuable insights into the impact of chronic stress on endocrine function, and these results are consistent with a substantial body of existing research. The observed elevation in cortisol levels in the morning, followed by a gradual decline throughout the day, mirrors findings from previous studies on altered diurnal cortisol patterns in individuals with chronic stress. For instance, research by MacLean (1997) [10] indicated that chronic stress is associated with disruptions in the normal cortisol secretion rhythm, a pattern also evident in this study. Moreover, the negative correlation between cortisol levels and thyroid hormones (T3 and T4) found in this study supports previous work by Guo *et al.* (2018) [11], who reported a reduction in T3 levels in patients with cardiovascular disease and elevated cortisol. This connection between cortisol and thyroid hormones highlights the intricate relationship between stress and thyroid function, reinforcing the idea that prolonged stress can suppress thyroid activity. The significant decrease in testosterone and estrogen levels observed in participants with high chronic stress aligns with prior research on the suppressive effects of stress on reproductive hormones. Hamilton *et al.* (2013) [12] found that chronic stress can disrupt the hypothalamic- pituitary-gonadal axis, leading to decreased levels of sex hormones, particularly in those with high stress levels. The results of this study, therefore, further emphasize that chronic stress can impact reproductive health by reducing sex hormone production. Additionally, this study's identification of thyroid-stimulating hormone (TSH) as a potential biomarker for chronic stress is in line with findings by Jonsdottir *et al.* (2019) [13], who suggested that TSH could serve as an important marker for assessing stress levels. In their research, they found a significant increase in TSH levels under stressful conditions, which supports the notion that TSH may reflect stress-induced changes in the endocrine system.

## Conclusion:

Chronic stress significantly impacts endocrine function, altering cortisol, thyroid, and sex hormone levels. These hormonal changes can lead to various health issues, highlighting the importance of identifying biomarkers for early detection and intervention.

## Figures and Tables

**Table 1:** Demographic characteristics of participants

**Characteristic**	**Frequency**	**Percentage (%)**
**Gender**		
Male	60	50
Female	60	50
Age (mean±SD)	34.5±8.7	
Chronic Stress Level		
Low Stress	30	25
Moderate Stress	60	50
High Stress	30	25

**Table 2:** Cortisol levels across different times of day

**Time of Day**	**Cortisol Level (µg/dL)**	**Mean±SD**	**P-value**
Morning	15.2±4.5	0.002	
Afternoon	9.4±3.8		
Evening	5.3±2.2		

**Table 3:** Thyroid hormone levels in participants with varying stress levels

**Stress Level**	**T3 (ng/mL)**	**T4 (µg/dL)**	**TSH (µIU/mL)**	**P-value**
Low Stress	1.5±0.3	7.8±1.2	2.1±0.5	0.14
Moderate Stress	1.4±0.4	7.5±1.4	2.3±0.6	0.04*
High Stress	1.2±0.5	6.9±1.5	2.8±0.7	

**Table 4:** Sex hormones in relation to chronic stress levels

**Stress Level**	**Testosterone (ng/dL)**	**Estrogen (pg/mL)**	**P-value**
Low Stress	500±75	180±50	0.02*
Moderate Stress	450±80	170±55	0.03*
High Stress	375±90	150±60	

**Table 5:** Correlation between chronic stress and hormonal changes

**Hormone**	**Stress Level (PSS Score)**	**Correlation Coefficient (r)**	**P-value**
Cortisol	High Stress (PSS > 26)	0.78	0.01*
T3	Moderate Stress (PSS = 16-25)	-0.54	0.03*
T4	High Stress (PSS > 26)	-0.62	0.01*
Testosterone	High Stress (PSS > 26)	-0.49	0.04*
Estrogen	High Stress (PSS > 26)	-0.51	0.03*
